# The Influence of Cold Temperature on Cellular Excitability of Hippocampal Networks

**DOI:** 10.1371/journal.pone.0052475

**Published:** 2012-12-31

**Authors:** Elvira de la Peña, Annika Mälkiä, Hugo Vara, Rebeca Caires, Juan J. Ballesta, Carlos Belmonte, Felix Viana

**Affiliations:** Instituto de Neurociencias de Alicante, Universidad Miguel Hernández-CSIC, San Juan de Alicante, Alicante, Spain; Universidade Federal do ABC, Brazil

## Abstract

The hippocampus plays an important role in short term memory, learning and spatial navigation. A characteristic feature of the hippocampal region is its expression of different electrical population rhythms and activities during different brain states. Physiological fluctuations in brain temperature affect the activity patterns in hippocampus, but the underlying cellular mechanisms are poorly understood. In this work, we investigated the thermal modulation of hippocampal activity at the cellular network level. Primary cell cultures of mouse E17 hippocampus displayed robust network activation upon light cooling of the extracellular solution from baseline physiological temperatures. The activity generated was dependent on action potential firing and excitatory glutamatergic synaptic transmission. Involvement of thermosensitive channels from the transient receptor potential (TRP) family in network activation by temperature changes was ruled out, whereas pharmacological and immunochemical experiments strongly pointed towards the involvement of temperature-sensitive two-pore-domain potassium channels (K_2P_), TREK/TRAAK family. In hippocampal slices we could show an increase in evoked and spontaneous synaptic activity produced by mild cooling in the physiological range that was prevented by chloroform, a K_2P_ channel opener. We propose that cold-induced closure of background TREK/TRAAK family channels increases the excitability of some hippocampal neurons, acting as a temperature-sensitive gate of network activation. Our findings in the hippocampus open the possibility that small temperature variations in the brain *in vivo*, associated with metabolism or blood flow oscillations, act as a switch mechanism of neuronal activity and determination of firing patterns through regulation of thermosensitive background potassium channel activity.

## Introduction

Homeothermic animals maintain a core temperature of 36–37°C within narrow ranges. Still, mammalian brain temperatures experience fluctuations of 1–3°C during physiological activities, such as exercise and feeding or in response to stressful stimuli [1], [2]. Even more pronounced changes in brain temperature occur during anesthesia or administration of psychoactive drugs [3]. In turn, changes in brain temperature have dramatic effects on neural function. For example, hyperthermia induces epileptiform-like activity in hippocampal slices [4], [5] and fever greatly increases the likelihood of epileptic discharges in children [6]. In contrast, a reduction in brain temperature precedes the onset of sleep, a brain state exhibiting a variety of characteristic activity patterns [7], [8].

Temperature affects the kinetics of chemical reactions, influencing basic neuronal properties such as single ion channel conductance, membrane input resistance, speed of action potential propagation and the time course of synaptic potentials [9], [10] Accordingly, cooling has been found to reversibly depolarize the membrane potential and increase the input resistance as well as the amplitude and duration of action potentials in hippocampal and cortical neurons [11]–[13]. Furthermore, decreasing temperature reportedly reduces the clearance of the excitatory neurotransmitter glutamate from the synaptic cleft [14] and diminishes the excitatory postsynaptic current (EPSC) amplitude [15]. Hippocampal field potentials are strongly temperature-sensitive, and temperature reductions diminish the field excitatory postsynaptic potential slope (EPSP) and increase the population spike amplitude [16]–[18]. The multiple effects of temperature on individual neuronal parameters result in complex consequences at the network level. For example, reduction of temperature results in a progressive prolongation of up states in cortical networks and non-linear changes in down-state duration, with a minimum at physiological temperatures [19]. Moreover, the recent discovery of several ion channels with high thermal sensitivity results in an added layer of molecular complexity, suggesting that their differential expression in particular neurons may influence their response to small temperature variations [20], [21]. In contrast to a wealth of knowledge about the function of different families of thermosensitive ion channels in the peripheral nervous system [22], [23] very little is known about the role of specific channels in the thermosensitivity of CNS neurons.

We investigated the influence of small temperature reductions around physiological values on the excitability of networks of hippocampal neurons in culture and postnatal hippocampal slices. We found a novel, unexpected increase in network activity mediated by closure of temperature-sensitive two-pore-domain potassium channels (KCNK), TREK/TRAAK channels [24]–[27].

## Materials and Methods

### Culture of Hippocampal Neurons

Hippocampal neurons from E17 mice were cultured as described previously [28]. In brief, pregnant Swiss OF1 mice were sacrificed by cervical dislocation. The hippocampus was isolated from the cerebrum of 17-day-old embryos; dissociated enzymatically with trypsin, followed by mechanical dissociation with a fire-polished Pasteur pipette; and the cells obtained were plated on poly-l-lysine-coated round coverslips (12 mm diameter) at 300.000 cells/coverslip. After 3 hours in Eagle’s minimal essential medium (MEM) with Earle’s salts, supplemented with 10% FBS; 4.5 g/l glucose; 1% GlutaMax; 1 mM Na-pyruvate; and 1% penicillin/streptomycin, the culture medium was changed to Neurobasal supplemented with 2% B-27, 1% GlutaMax and 1% penicillin/streptomycin. Half of the culture medium was exchanged every 3–4 days, and the cells were recorded after 9–11 days in culture.

All experimental procedures were carried out according to the Spanish Royal Decree 1201/2005 and the European Community Council directive 2007/526/EC. The ethics committee from Universidad Miguel Hernández, Alicante, Spain, approved this study.

### Electrophysiology in Cultured Neurons

Cell-attached and whole-cell voltage- or current-clamp recordings were performed simultaneously with temperature recordings. Pyramidal-shaped neurons were patched randomly in the dish. The bath solution contained (in mM): 140 NaCl, 3 KCl, 1.3 MgCl_2_, 2.4 CaCl_2_, 10 HEPES, 10 glucose, pH 7.4_._ Standard patch-pipettes (5–7 MΩ) were made of borosilicate glass capillaries (Harvard Apparatus Ltd, UK). As the age of our hippocampal cultures coincides with the gradual decrease in the intracellular chloride concentration during neuronal development [29], , we tested two different pipette solutions with different chloride concentrations. The high Cl^-^ solution (HCS) contained (in mM): 140 KCl, 10 NaCl, 4 Mg-ATP, 0.4 Na-GTP, 10 HEPES. The low Cl^−^ solution (LCS) contained (in mM): 154 K-gluconate, 4 NaCl, 0.5 MgCl_2_, 4 Mg-ATP, 0.4 Na-GTP, 10 HEPES. In both solutions, osmalarity was 300 mOsm/kg and the pH 7.3, adjusted with KOH. The solution used for individual neurons is identified in the figure legend. Signals were acquired with a patch-clamp amplifier (Axopatch 200B; Molecular Devices, Sunnyvale, USA or EPC-8; Heka Elektronik, Lambrecht/Pfalz, Germany). Data were digitized with an analog-to-digital converter (Digidata 1322; Molecular Devices, Sunnyvale, USA). Stimulus delivery and data acquisition were performed using pCLAMP software (versions 8–9, Molecular Devices, Sunnyvale, USA). The liquid junction potential was calculated using pCLAMP software and corrected.

### Slice Recordings

Mice were killed by cervical dislocation and the brain was quickly removed from the skull. For experiments recording evoked synaptic activity (age P30–P60), hippocampi were dissected in cold standard artificial cerebrospinal fluid (ACSF), transverse slices (400 µm) were cut with a manual tissue chopper and placed in a submerged holding chamber for at least 1 hour at room temperature (RT). For experiments recording hippocampal spontaneous activity (age P15–P21), horizontal brain slices (400 µm) were cut in cold ACSF using a Vibratome (Pelco ,TPI, series 1000, St. Louis, USA), and placed in a humidified interface holding chamber for at least 1 hour at RT. Storing of slices in interface chambers helps preserve the network integrity required for the generation of sharp wave-ripple (SPW-R) complexes [31]. The composition of the standard ACSF was (in mM): NaCl 120, KCl 2.5, NaH_2_PO_4_ 1.0, MgCl_2_ 1.2, CaCl_2_ 2.5, NaHCO_3_ 26.2, glucose 11, pH 7.4 when equilibrated with carbogen (95% O_2_ - 5% CO_2_). For recording, individual slices were transferred to a submerged chamber and superfused with ACSF at 36–37°C at a constant rate (2–3 ml/min). The temperature of ACSF in the bath was adjusted with a water-cooled Peltier device.

Extracellular activity was recorded with glass micropipettes (4–6 MΩ) filled with standard ACSF and coupled to the input stage of a Multiclamp 700B amplifier (Molecular Devices, Sunnyvale, USA). Signals were amplified 1000-fold and sampled at 10 kHz with a Digidata 1440A converter. Data were collected using pClamp10 software (Molecular Devices, Sunnyvale, USA).

Spontaneous SPW-R activity was recorded in stratum pyramidale of the CA3 area, where it has been described to emerge and propagate towards CA1, both *in vivo* [32] and *in vitro* [31]. A modified, Mg-free ACSF was used to favour the incidence of SPW-R activity. Its composition was as follows (in mM): NaCl 129, KCl 5, NaH_2_PO_4_ 1.25, CaCl_2_ 1.6, NaHCO_3_ 21, glucose 10. In addition, bicuculline methiodide (BMI) 10 µM was also added to induce the appearance of short bursts of epileptiform ictal activity after SPW-R complexes [33].

Extracellular evoked field potentials were recorded in stratum radiatum of the CA1 area. Stimuli (0.1 ms pulse width) were delivered to the Schaffer collateral /commissural afferents at 0.2 Hz through a concentric bipolar stainless steel electrode using a constant current isolated stimulator (Digitimer, Welwyn Garden City, UK).

### Temperature Stimulation

Coverslip pieces with cultured cells were placed in a microchamber and continuously perfused with solutions warmed at 36–37°C, from hereon referred to as baseline temperature. The temperature was adjusted with a water-cooled Peltier device placed at the inlet of the chamber and controlled by a feedback device. Cold-sensitivity of the electrical activity was tested during transient temperature drops of the bath solution.

### Western Blot

For immunoblot analysis hippocampal cell cultures, 9–11 DIV, were removed with RIPA buffer composed of 150 mM NaCl, 50 mM Tris–HCl pH 7.4, 1% Nonidet P-40 (v/v), 1 mM EDTA, 0.25 mM sodium deoxycholate, and the Complete Mini® protease inhibitor cocktail (Roche Applied Science, Indianapolis, USA). Mouse hippocampal tissue was obtained from P10 animals, and were minced on ice and homogenized in RIPA. Tissue and cultured hippocampal cells samples were sonicated on ice for 5 min, centrifuged at 10.000 rpm and 4°C for 10 min, and the supernatant was collected for protein analysis using a BCA assay kit (Thermo Fisher Scientific, Rockford, USA), 20 µg of protein was loaded per lane. Samples were mixed with sample buffer (4% SDS (w/v), 10% glycerol (v/v), 4% 2-mercaptoethanol, 0.2% bromophenol blue (w/v), and 50 mM Tris–HCl, pH 6.8), heated at 95°C for 5 min, and then subjected to SDS-PAGE gel electrophoresis (10% acrylamide gels).

Proteins were transferred (semi dray transfer) onto a nitro-cellulose membrane Hybond™ ECL (Amersham Biosciences, Piscataway, USA). Membranes were blocked with 5% non-fat milk in TBS-Tween (Tris-buffered saline and 1% Tween-20), and subsequently incubated, overnight at 4°C, with the primary polyclonal antibodies, TREK-1 (APC-047),TREK-2 (APC-055) ,TRAAK(APC-108), from Alomone Labs (Jerusalem, Israel), at 1∶200 dilution. To avoid cross-reaction, antiTREK-1 was pre-incubated with microsomal fraction of transfected HEK293 cells expressing TREK-2 and TRAAK during 1 h at room temperature. Thereafter the sample was centrifuged and the supernatant was collected to detect expression of TREK1 in membranes. To detect TREK-2 antiTREK-2 was pre-incubated with microsomal fraction of transfected HEK293 cells expressing TREK-1 and TRAAK. To detect TRAAK antiTRAAK was pre-incubated with microsomal fraction of transfected HEK293 cells expressing TREK-1 and TREK-2.

Blots were treated with peroxidase-conjugated rabbit anti-goat IgG at 1∶2000 dilution for 1 h at room temperature, and protein signals were revealed using the ECL Advance™ Western Blotting Detection Kit (Amersham Biosciences. Piscataway, USA). Finally, bands were digitalized and quantified using a LAS-1000 Bioimager (Fujifilm Co., Barcelona, Spain) and data analysis was performed using the Image Gauge 4.0 software (Fujifilm Co.).

The specificity of the anti-TRAAK antibody was tested in TRAAK KO mice, generously provided by M. Lazdunnski [34].

### Chemicals

Baclofen was from Research Biochemicals Inc. (Natick, USA) while the remaining synaptic transmission modulators 6-cyano-7-nitroquinoxaline-2,3-dione (CNQX), dl-2-amino-5-phosphonopentanoic acid (AP-V), (−)bicuculline methiodide (BMI), as well as the glutamate transporter blocker dl-threo-*β*-benzyloxyaspartate (TBOA) and tetrodotoxin citrate (TTX) were purchased from Tocris (Tocris Bioscience, Bristol, UK), and were stored as aqueous stock solutions at −20°C. The TRP channel modulators l-menthol (Scharlau, Spain), allyl isothiocyanate (AITC), ruthenium red (RR) and capsaicin (all from Sigma); 4-[3-chloro-pyridin-2-yl]-piperazine-1-carboxylic acid [4-tert-butyl-phenyl]-amide (BCTC, Grünenthal GmbH, Aachen, Germany); [2-(1,3-dimethyl-2,6-dioxo-1,2,3,6-tetrahydro-7H-purin-7-yl)-*N*-(4-isopropylphenyl)-acetamide] (HC030031, Hydra Biosciences, Cambridge, USA); and the 2-pore potassium channel modulators nifedipine (Research Biochemicals Inc. Natick. USA), amlodipine (Enzo Life Sciences Ltd., Exeter, UK) arachidonic (Sigma-Aldrich) acid and riluzole hydrochloride (Sigma-Aldrich) were stored at −20°C as ethanol or dimethyl sulfoxide (DMSO) stock solutions. Fresh dilutions of all chemicals were prepared prior to the experiments. Chloroform (CHCl_3_) was from Merck, and its working solution was prepared immediately before application.

### Data Analysis

Data from electrophysiological recordings were sampled at 10 kHz and filtered online at 2 kHz with Clampex, (Molecular Devices, Sunnyvale, USA) stored, and analyzed offline with a computer using the Event Detection application of Clampfit (pCLAMP 9.0, Molecular Devices, Sunnyvale, USA). Unless noted otherwise, events were analyzed from baseline-corrected, 120 Hz lowpass-filtered traces. Baseline events were detected as those occurring spontaneously at 36–37°C with a threshold of 3–4 standard deviations of the noise. Specific detection of cooling-evoked large currents was performed setting the threshold at a level excluding the baseline events. Data are reported as mean±standard error of the mean. When comparing two means, statistical significance (p<0.05) was assessed by Student’s two-tailed *t*-test. For multiple comparisons of means, one- or two-way repeated measures ANOVA followed by post-hoc Dunnett’s or Tukey’s tests were performed using GraphPad Prism version 4.00 for Windows (GraphPad Software, San Diego, USA).

## Results

### Cooling Increases Electrical Activity of Cultured Hippocampal Networks

**Figure 1:**
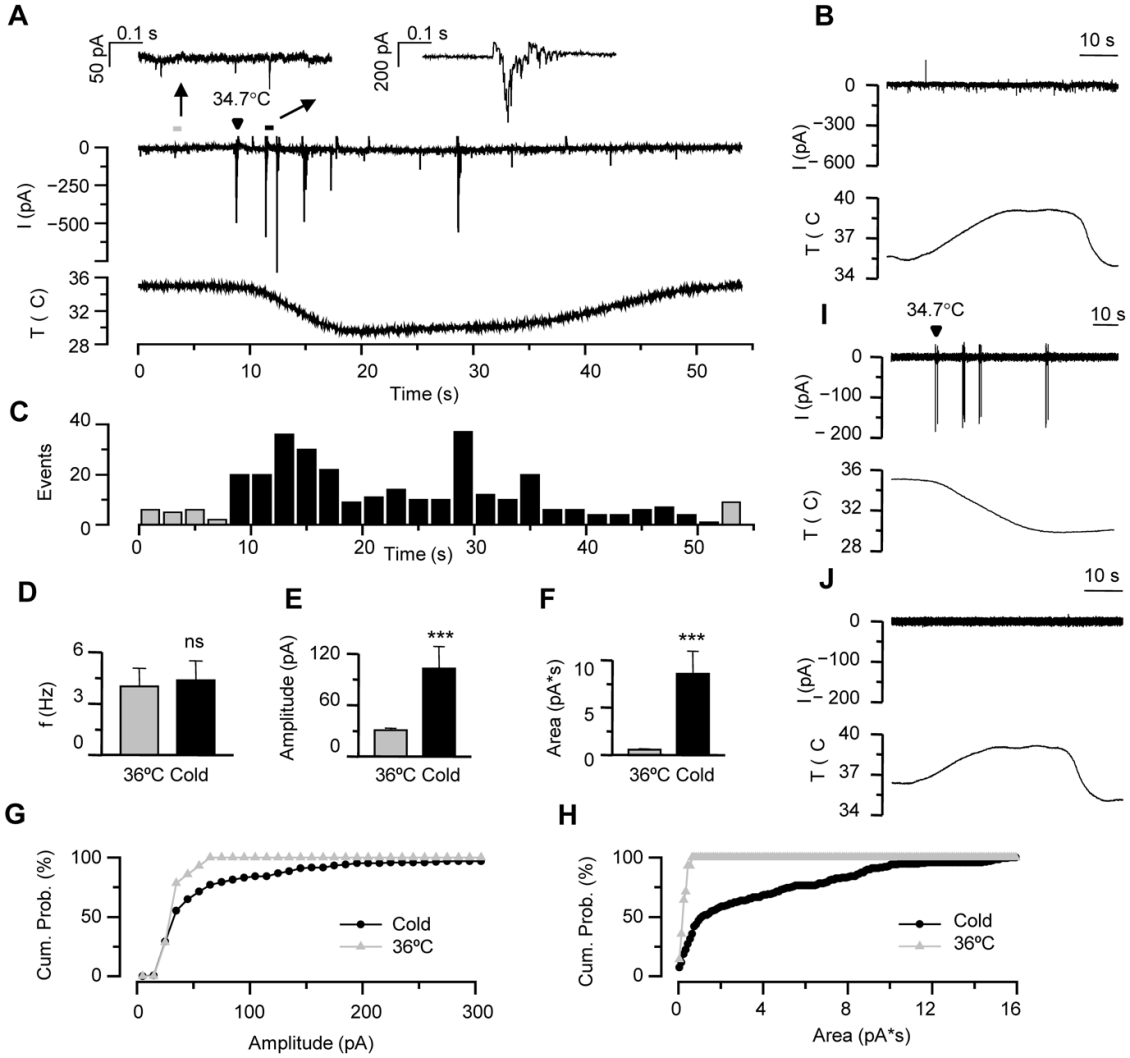
Moderate cooling evokes large current responses in hippocampal neurons. *A*, Time course of whole-cell current at −60 mV in a hippocampal neuron subjected to a cooling ramp from 35°C. Insets show individual current events (marked with an arrow) at baseline temperature and during the initial cooling period on an expanded time scale. The arrowhead marks the occurrence and temperature threshold of the first cold-evoked event. *B*, Response of the same neuron to heating from 35°C to 39°C and subsequent cooling. Note the absence of response during heating or during cooling from a higher baseline value. *C*, Number of events during cooling ramp for the neuron shown in A quantified in 2-second bins. *D*, Mean event frequency, *E*, mean amplitude and *F*, mean area during basal and cooling conditions (n = 15). Statistical significance in panels D–F was assessed with Student’s paired *t*-test (*p<0.05; **p<0.01. *G*–*H*, Cumulative probability histogram of event amplitude (*G*) and event area (*H*) at 35°C *vs.* during cooling of the recording shown in A. Note the tendency of cooling to shift both event amplitude and area towards larger values. *I*–*J*, Time course of cell-attached action currents recorded in the same hippocampal neuron, during identical cooling and heating protocol. Note the very similar characteristics of threshold and pattern as recorded in the whole-cell configuration. All recordings in this figure were performed in LCS (see Methods).

All hippocampal neurons studied exhibited spontaneous synaptic activity at 35–36°C when recorded in whole-cell voltage-clamp configuration at a holding potential of −60 mV (Figure 1A). The synaptic currents consisted of brief excitatory and/or inhibitory miniature postsynaptic currents (mIPSC and mEPSC). As seen in Figures 1A and 1C, cooling of the perfusion solution (from 35°C to 29°C) evoked large transient currents of both inward and outward nature. The effect of cooling was a marked increase in the mean amplitude and mean area of the events, while the frequency was not significantly altered as seen in Figures 1D–F (n = 15). Accordingly, cumulative probability histograms of the events before and during the cooling ramp show how cooling shifts event amplitude and area to larger values (Figures 1G–H). The lack of effect of cooling on overall event frequency is explained by two factors. First, cold-evoked events are relatively sparse (i.e. low frequency) compared to basal, spontaneous activity. Second, cold-evoked activity tends to decline during sustained cooling. Changing the intracellular recording solution from low chloride [LCS], typical of mature neurons, to a higher chloride concentration [HCS], characteristic of immature neurons [29], [30], shifted the balance of inward and outward events towards the former, but had no effect on the occurrence of the cooling-evoked large amplitude responses (not shown).

The threshold of the cooling-evoked events in whole-cell voltage clamp, defined as the onset of the large amplitude responses, was 34.3±0.4°C (n = 14). As illustrated for the same neuron, heating (Fig. 1B) or displacing the cooling ramp to higher temperatures (i.e. cooling from 39°C to 35°C) (Fig. 1B) did not produce an increase in synaptic activity, suggesting that the observed phenomenon has a fixed threshold and is not a result of temperature change *per se*. As seen in Figure 1I, the cooling-activated responses were also observed as large action currents when the same cell was recorded in the cell-attached configuration, indicating that they are not secondary to changes in the cytoplasmic content or due to cell damage. Again, heating or displacing the cooling ramp to higher temperatures did not evoke spiking activity (Fig. 1J), and the threshold temperature of the cooling-evoked action currents was very similar to that determined in whole-cell configuration, 33.6±0.6°C (p = 0.3, n = 12, unpaired *t*-test).

Altogether these results show the emergence of a novel class of electrical activity in the hippocampal network during a modest drop in temperature that is characterized by large amplitude current transients.

### Cooling-evoked Responses are Synaptically Mediated

**Figure 2:**
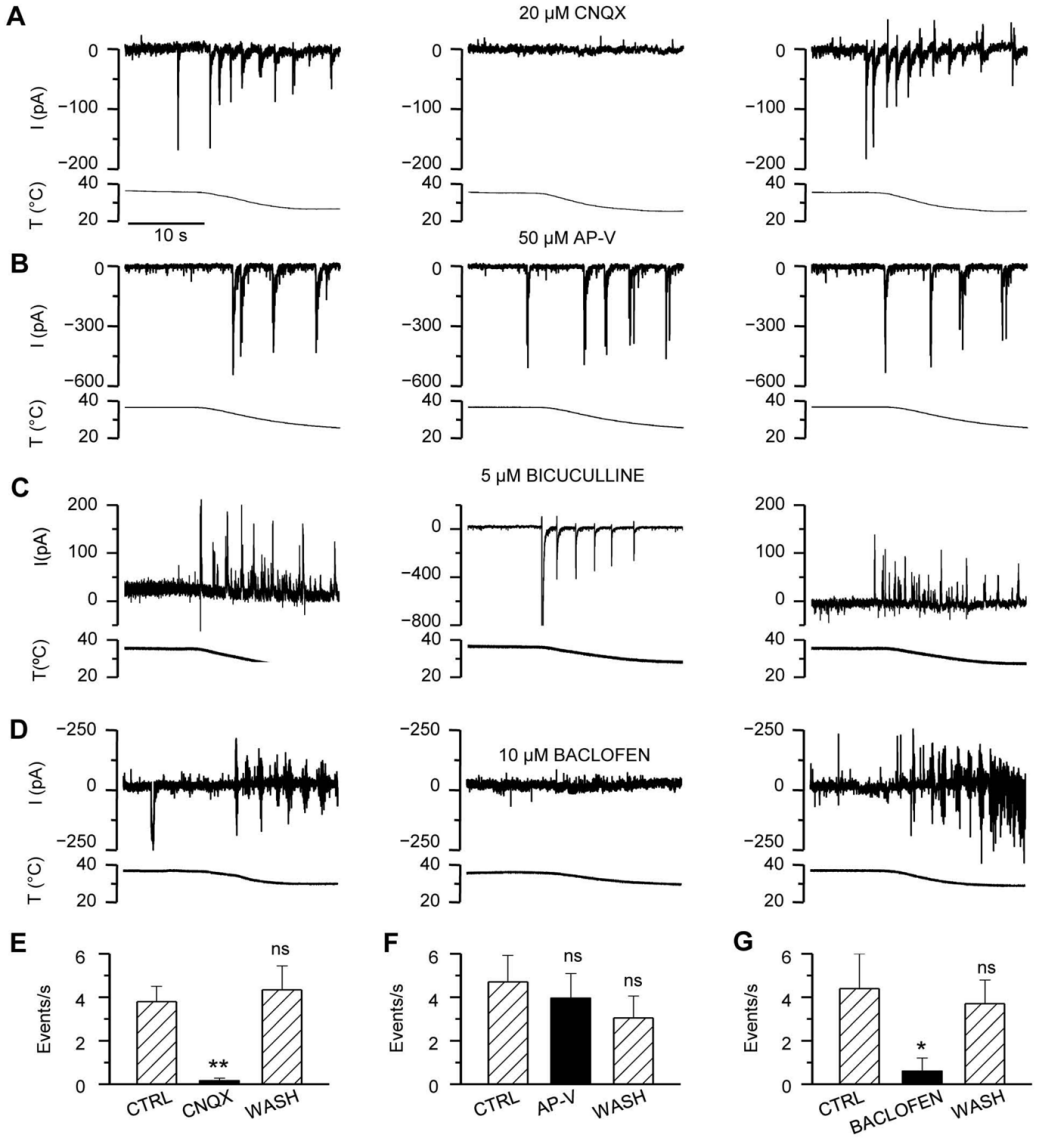
Differential effect of synaptic blockers on cooling-evoked responses. *A*–*D*, Time courses of whole-cell current at a holding potential of −60 mV in four hippocampal neurons in the absence and presence of the synaptic blockers *A*, CNQX; *B*, AP-V; *C*, Bicuculline; *D*, Baclofen. *E*–*G*, Summary histogram of the number of events during cooling in the absence and presence of *E*, CNQX (n = 5); *F*, AP-V (n = 4); and *G*, Baclofen (n = 4). Statistical significance in panels D–F was assessed with repeated-measures 1-way ANOVA in combination with Dunnett’s post-test with respect to the first cooling stimulus in control conditions, and indicated with: *p<0.05; **p<0.01. All recordings, except B, were obtained in LCS. Time scale shown in A, applies to all traces (A–D).

Next, we wished to establish whether neurotransmitter release is involved in the observed electrical activity. CNQX is an antagonist of AMPA- and kainate-sensitive excitatory glutamate receptors. As seen in Figures 2A and 2E, 20 µM CNQX abolished the inward synaptic activity at 36–37°C as well as the cooling-evoked large transient inward events; on a few occasions some outward components remained during cooling. The effects of CNQX were fully reversible upon wash. We did not observe any CNQX-induced increase in the frequency of spontaneous GABA_A_ receptor-mediated postsynaptic currents (sIPSCs) as reported in some studies using slice preparations [35].

In contrast, application of 50 µM AP-V had no effect on the cooling-evoked synaptic currents, suggesting a minor contribution of NMDA glutamate receptors to the observed responses (Figure 2B, 2F). As expected, 5 µM bicuculline, a blocker of inhibitory GABA_A_ receptors, eliminated all baseline and cooling-evoked outward responses, while cooling-evoked inward events remained. Moreover, in those recordings, in which cooling-evoked activity was of outward nature only, the bicuculline-induced absence of inhibitory input from GABA_A_ receptors led to the appearance of large, cooling-evoked inward events (Figure 2C). In several neurons, the application of bicuculline generated a large epileptiform-like excitatory activity that obscured other activity patterns (data not shown).

Baclofen activates presynaptic GABA_B_ receptors, downregulating excitatory transmitter release [36]. GABA_B_ receptors are also known to modulate postsynaptic G-protein-coupled inwardly rectifying potassium (GIRK) channels [37] and N- and P/Q-type calcium channels, that are critical for calcium entry during neurotransmission [38]. As seen in Figures 2D and 2G, in the presence of 10 µM baclofen the cooling-evoked discharges were fully suppressed, further confirming that these responses result from an excitatory synaptic activation of the neuronal network. Taken together, these results demonstrate that the large current transients appearing during cooling rely on synaptic transmission within the neuronal network.

### Current-clamp Recordings Confirm Membrane Depolarization and the Synaptic Nature of the Responses to Cold

**Figure 3:**
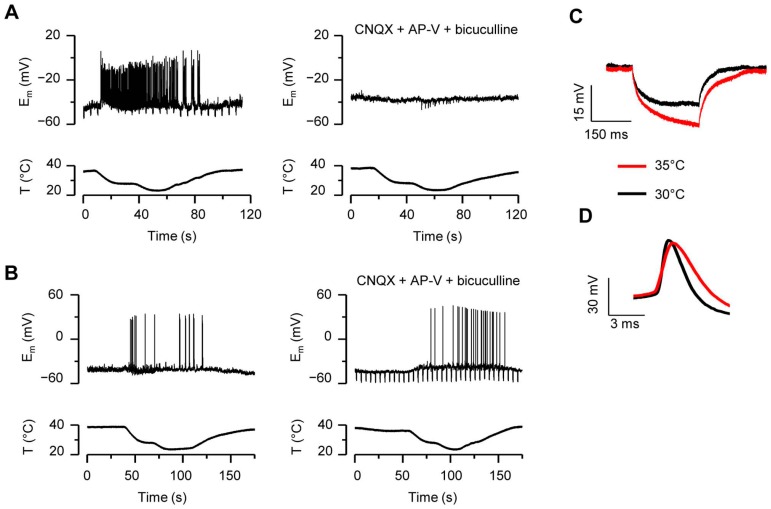
Hippocampal neurons fire action potentials in response to cooling. *A–B*, Time course of membrane potential change of two hippocampal neurons recorded in whole-cell current-clamp mode at −60 mV showing the cooling-evoked firing of action potentials. Note how the response of the neuron in A is completely abolished in the presence of a cocktail of synaptic blockers (20 µM CNQX+50 µM AP-V+5 µM bicuculline), whereas the neuron in B continues firing. The vertical lines in some of the voltage traces correspond with a pulse protocol for determination of membrane resistance. *C–D*, Effect of temperature on electrophysiological properties in current-clamp mode in the presence of a cocktail of synaptic blockers *C*, Membrane resistance was obtained from the membrane potential change in response to a 25 pA pulse of 250-millisecond-duration. *D,* Action potentials were evoked at rheobase using depolarizing current pulses of 450-ms-duration at a membrane potential of −60 mV. The neuron shown in C–D was silent in the presence of synaptic inhibitors. All recordings in [LCS].

We performed current-clamp recordings at −60 mV in 25 neurons and found that in the absence of synaptic blockers, all of them responded to cooling with robust action potential firing (Figures 3A–B). When the same neurons were subjected to a cooling ramp in the presence of a cocktail of synaptic blockers (CNQX 20 µM+AP-V 50 µM+bicuculline 5 µM), no action potentials were observed in 20 neurons, while 5 of them maintained the spiking activity (Figures 3A–B). No differences between the resting membrane potential of these two groups of neurons were found (−59±1 mV n = 20 *vs.*; −58±1 mV n = 5; p = 0.95, unpaired *t*-test). In neurons spiking in presence of synaptic blockers, cooling produced a progressive depolarization of the membrane potential (4.0±0.7 mV at 30°C; 8.3±1.3 mV at 20°C; n = 5). In contrast, neurons that were silent in the presence of synaptic blockers hyperpolarized slightly upon cooling (−2.5±1.1 mV at 30°C; −0.6±1.7 mV at 20°C; n = 20). The depolarization analyzed in the presence of synaptic blockers to avoid excessive fluctuations due to action potentials, was statistically different between the two groups (p<0.001, unpaired *t*-test). Cooling also increased the input resistance of the neurons but no differences were found between firing and silent neurons (17±5%, n = 5 *vs.* 21±11%, n = 13, increase in resistance for a temperature drop to 30°C; p = 0.5, unpaired *t*-test). While neurons firing in the presence of blockers tend to exhibit a higher mean threshold temperature than silent neurons (33.9±1.3°C *vs.* 31.2±0.7°C), the difference was not statistically significant (p = 0.1, n = 5 *vs.* n = 20). Similarly, the threshold temperature appeared to decrease when the neurons were recorded in the presence of blockers (33.5±1.3°C in control solution *vs.* 31.3±1.9°C in blockers, p = 0.3, paired *t*-test, n = 5), but the difference lacks statistical significance.

We also analyzed the effect of modest temperature reductions on basic electrophysiological properties of 8 hippocampal neurons recorded in current-clamp whole-cell configuration, in the presence of the cocktail of synaptic blockers, to prevent cold-evoked firing (Table 1). Two additional neurons continued to fire action potentials in response to cooling in the presence of synaptic inhibitors. The mean resting potential of the firing and silent neurons were very similar (−60±6 mV *vs.* −65±3 mV for firing and silent neurons, respectively). Moreover, no statistical differences were found between the remaining electrophysiological characteristics of these two groups of neurons at 35°C (not shown). As seen in Figures 3C–D, the effect of cooling was to increase the input resistance and the spike duration of the neurons [11], [13], while the spiking frequency to a depolarizing current pulse was slightly reduced. Likewise, depolarization and repolarization rates of the action potential were slower at 30°C compared to 35°C.

**Table 1:** Effect of temperature on the electrophysiological properties of hippocampal neurons recorded in current-clamp configuration in the presence of synaptic blockers (20 µM CNQX+50 µM AP-V+5 µM bicuculline), which in these abolished cooling-evoked action potential firing.

	Resistance(MΩ)		Rheobase(pA)		Threshold(mV)		Spike amplitude (mV)		Spike duration(ms)		dV/dtmax		dV/dtmin		Spike frequency (Hz)	
	*35°C*	*30°C*	*35°C*	*30°C*	*35°C*	*30°C*	*35°C*	*30°C*	*35°C*	*30°C*	*35°C*	*30°C*	*35°C*	*30°C*	*35°C*	*30°C*
Mean	263	323^b^	35	26	−43	−42	53	48	1.7	2.6	66	45^a^	32	21	19	18
SEM	40	53	9	6	2	2	4	5	0.3	0.6	12	11	6	5	2	2

The parameters were obtained using depolarizing current pulses of 450-ms-duration at a membrane potential of −60 mV. Membrane resistance was calculated from the I–V slope generated from depolarizing and hyperpolarizing pulses. Statistical significance between parameters obtained at 30°C *vs.* 35°C was determined with 2-way repeated-measures ANOVA, and indicated with ^a^ p<0.05; ^b^ p<0.001; n = 8 for all columns.

### Glutamate Spillover is not the Cause of the Cooling-evoked Events

**Figure 4:**
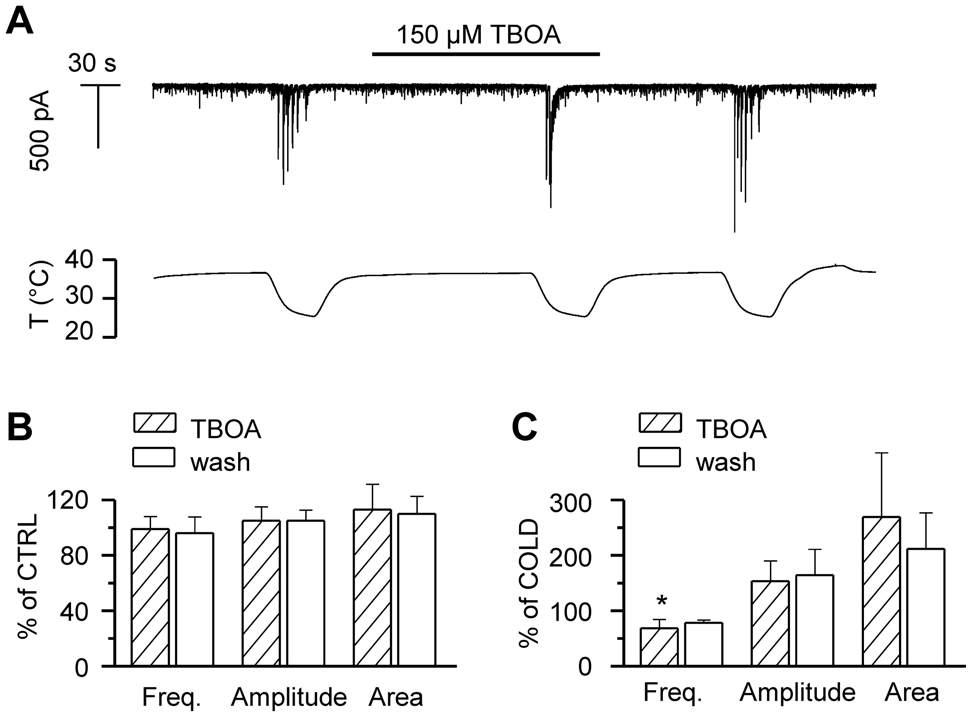
Glutamate spillover does not generate the cooling-evoked responses. *A*, Time course of whole-cell current [HCS] at −60 mV in a hippocampal neuron subjected to cooling ramps in the absence and presence of 150 µM DL-TBOA. Note, that TBOA does not affect synaptic activity at baseline temperature. *B,* Summary histogram of mean frequency, amplitude and area of synaptic events at baseline temperature 36–37°C. C, Summary histogram of mean frequency, amplitude and area of cooling-evoked events. In panels B–C, parameters in the presence of TBOA and during washout are normalized to data prior to TBOA application. Statistical significance was assessed with 1-way-ANOVA in combination with Dunnett’s post test with respect to the data prior to TBOA application, and is indicated with *p<0.05.

Clearance of the excitatory transmitter glutamate from the extracellular space is more efficient at higher temperatures [14]. Therefore, at lower temperatures glutamate spillover is expected to occur at the synaptic cleft, thus increasing cross-talk between neighbouring synapses. dl-TBOA, an antagonist of the various types of excitatory amino acid transporters, EAATs [39], delays the clearance of glutamate, and increases the amplitude of NMDA and AMPA receptor-mediated responses. We applied 150 µM TBOA at 36–37°C to test whether we could reproduce the large current transients observed during cooling, by reducing the rate of glutamate transport at the synaptic cleft. Figure 4A shows the time course of the membrane current in a hippocampal neuron recorded in whole-cell voltage-clamp configuration at –60 mV, in the absence and presence of TBOA. Applied at baseline temperature, TBOA failed to generate the large synaptic currents induced by cooling. Event analysis (Figure 4B) showed that at 36–37°C, the frequency, amplitude and area of the synaptic events were not altered by TBOA. When TBOA was applied during cooling, in part of the experiments the large cooling-evoked events were grouped into fewer and larger discharges (Figure 4A), likely due to accumulation of glutamate, whereas in other recordings, such effect was not observed. Altogether, the mean frequency of events during cooling with TBOA was slightly reduced compared to the situation in control condition, while increases in mean amplitude and area were not statistically significant due to large variability (Figure 4C). Thus, we can rule out temperature-induced delayed glutamate clearance as the cause of the large synaptic discharges observed during cooling.

### TTX Abolishes Cold-evoked Synaptic Currents

**Figure 5:**
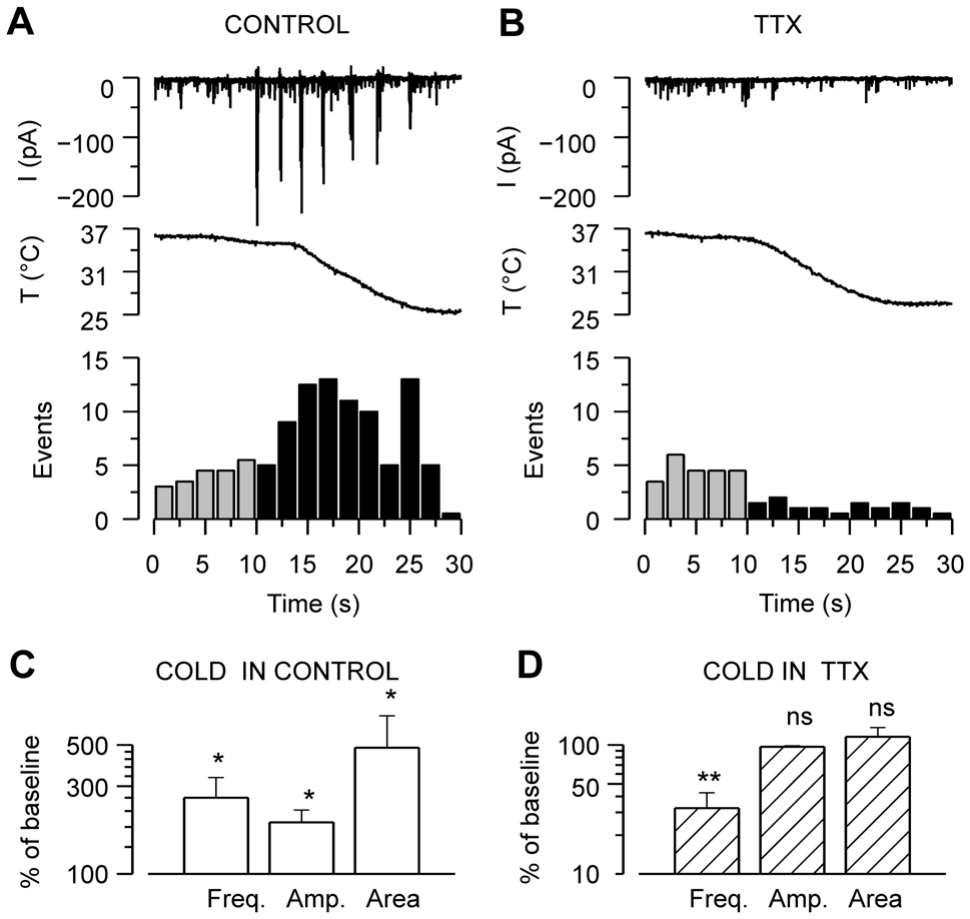
Action potential firing is required for cooling-evoked responses in the neuronal network. *A*–*B*, Time courses of whole-cell current [LCS] at a holding potential of −60 mV in a hippocampal neuron in the *A*, absence and *B*, presence of 1 µM TTX before and during cooling. Below, events detected in the same recordings are quantified in 2-second bins. *C*–*D*, Mean frequency, mean amplitude, and mean area of the synaptic currents of hippocampal neurons during cooling in *C*, control solution and *D*, TTX. Parameters during cooling are represented as percent of the values at baseline temperature. Note that in the absence of TTX, cooling increases all the synaptic event parameters, while in the presence of TTX, event frequency is reduced and the remaining parameters are unchanged. Statistical significance in panels C–D was assessed between each parameter during cooling and at baseline temperature with Student’s paired *t*-test: *p<0.05; **p<0.01, n = 6.

The strong dependence of cold-evoked suprathreshold events on intact synaptic connectivity (Figure 3A), together with the observed increase in membrane resistance and depolarization, prompted us to study the role of action potential firing in the observed synaptic responses. Application of 1 µM TTX, to block propagated action potentials, did not affect the baseline synaptic activity at 36–37°C, but completely abolished the large discharges (large inward, outward or mixed postsynaptic currents) during cooling (Figure 5A–B). When six neurons were cooled in the absence of TTX, their synaptic events increased on average 160% in frequency, 90% in amplitude and 380% in area from baseline values (Figure 5C). When the same cells were cooled in the presence of TTX, events amplitude and area were unaffected, whereas their frequency decreased to 30±10% of control (Figure 5D).

The effect of TTX implies an involvement of propagated action potentials in the cold-evoked responses, and together with the mixed nature of the discharges suggests the overall activation of the neuronal network. The reduction of event frequency during cooling in the presence of TTX reveals that the cold-evoked responses are unlikely to be caused by a summation effect of spontaneous mini-PSCs. Therefore, the cooling-evoked large synaptic responses seem to constitute a new phenomenon, independent of the spontaneous activity. From hereon, spontaneous activity (mini-PSCs) is analyzed separately from the cooling-evoked large amplitude responses.

### Cooling-induced Synaptic Currents are not Mediated by Activation of thermoTRPs

We explored the involvement of temperature-sensitive ion channels on the increased excitability of the hippocampal neuronal network, action potential firing and large synchronous synaptic discharges induced by cooling. First, candidates were sought from the thermosensitive members of the TRP family of non-selective cation channels [22]. Cold-sensitive TRP channels TRPA1 and TRPM8 are not present in primary cell cultures of hippocampus [28] but several reports suggest the hippocampal expression of various of the heat-sensitive TRPV channels [40]–[43]. Of them, TRPV3 and TRPV4 are active within the physiological temperature range [44]. The cooling-induced closing of a TRPV channel would cause hyper- and not depolarization of the cell membrane. However, the hyperpolarization of inhibitory interneurons could function as a trigger for subsequent events that would lead to membrane depolarization and the firing of action potentials in pyramidal cells.

**Figure 6:**
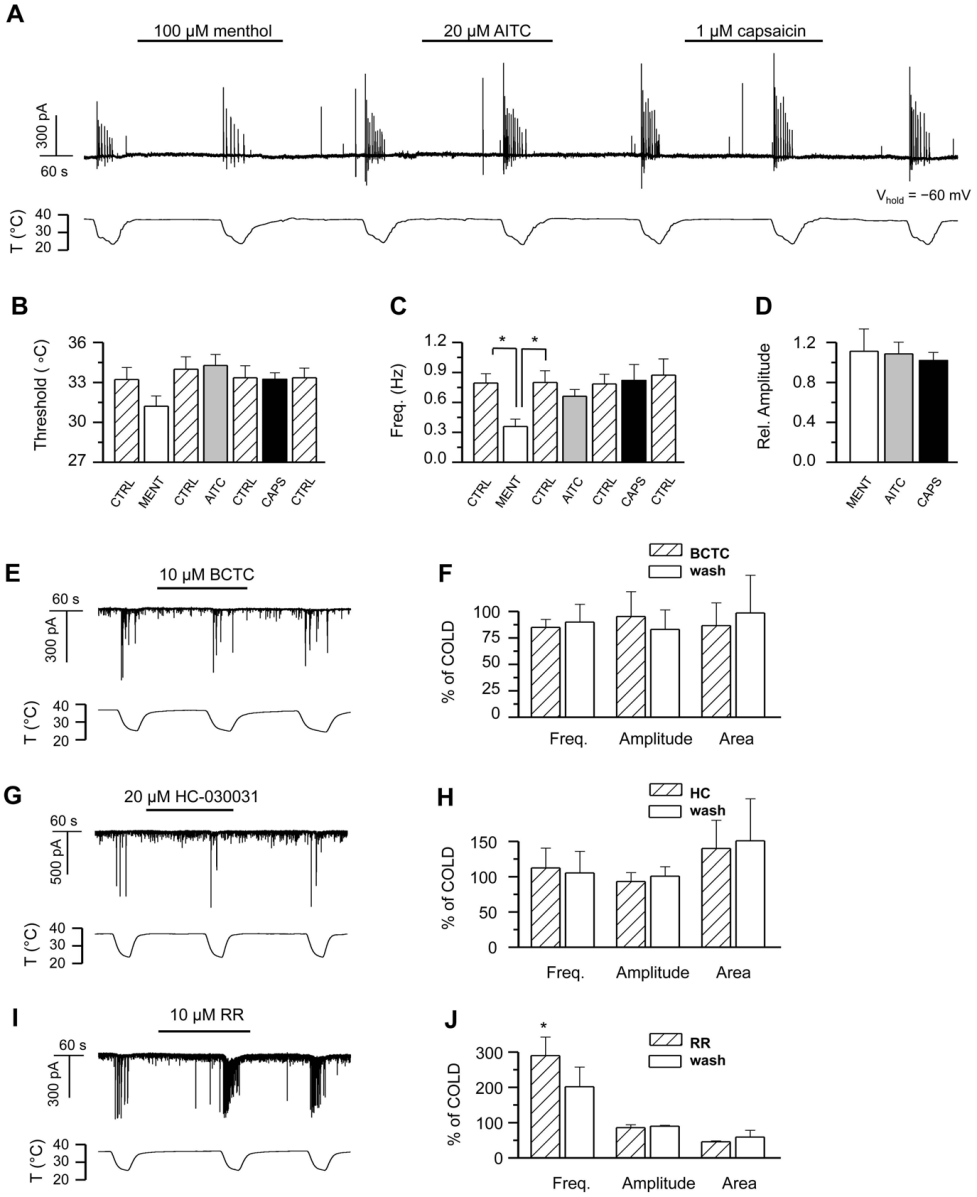
Temperature sensitive TRP channels are not involved in generating the cooling-evoked responses. *A*, Time course of whole-cell current at −60 mV in a hippocampal neuron during repetitive cooling ramps in the absence and presence of thermo-TRP agonists menthol (100 µM), AITC (20 µM) and capsaicin (1 µM). The time scale bar indicates the zero current level. *B*−*D,* Summary histograms showing the average effect of the agonists on the *B,* threshold, *C*, average frequency, and *D,* relative amplitude of the temperature-induced responses during the descending part of a cooling ramp (n = 5). *E*, Time course of whole-cell current at −60 mV in a hippocampal neuron during cooling ramps in the absence and presence of the thermo-TRP antagonist BCTC (10 µM); *F,* Summary histogram showing the effect of BCTC on the mean frequency, amplitude and area of the cooling-evoked responses, n = 3. *G*−*H*, 20 µM HC-030031, n = 4; *I*−*J,* 10 µM ruthenium red, n = 2. In panels F, H, J, parameters in the presence of antagonist and during washout are normalized to data prior to antagonist application. In the different panels, statistical significance was assessed with repeated-measures 1-way-ANOVA in combination with Tukey’s post test, and is indicated with *p<0.05 where applicable. All records shown were obtained in HCS except the neuron in panel A.

Thus, we explored the effect of specific agonists and antagonists of thermo-sensitive TRP channels on hippocampal cell cultures. Figure 6A shows the time course of the whole-cell current recorded in a hippocampal neuron during consecutive cooling ramps in the absence and presence of the specific agonists of TRPM8, TRPA1, and TRPV1, menthol (100 µM), AITC (20 µM), and capsaicin (1 µM) respectively [45]–[47]. These agonists had no effect on the characteristics of the baseline synaptic activity at 37°C (not shown). Analysis of the descending part of the cooling ramps revealed no statistically significant differences between event threshold and amplitude of the cooling-evoked responses in control solution *vs.* agonists (Figures 6B−D). The average event frequency of the cooling-evoked activity was likewise unaffected, except for the case of menthol, where its value was reduced 55±9% compared to control, contrary to what would be expected for activation of TRPM8 or TRPA1 channels. This reduction most likely results from a direct activation by menthol of inhibitory GABA_A_ receptors in hippocampal neurons in culture [48], thus augmenting the inhibitory component of neuronal activity regulation.

Next we tested different blockers of thermoTRP channels. BCTC is a blocker of TRPM8 and TRPV1, while it potentiates the responses of TRPA1 [49]–[51]. When we applied a saturating concentration of BCTC to the hippocampal cell cultures, no effect was observed on the baseline synaptic activity or the cooling-evoked responses (Figures 6E–F). Similarly, HC-030031, a specific antagonist of TRPA1 [52], failed to produce any change in the synaptic activity at baseline temperature or during cooling (Figures 6G–H), thereby ruling out cold-sensitive TRP channels and TRPV1 as generators of the cooling-evoked activity. In the presence of ruthenium red (RR), an inhibitor of the remaining thermoTRPs TRPV1–V4, as well as TRPA1[45], [53]–[56], the amplitude and area of the synaptic events remained unaffected both at baseline temperature and during cooling, whereas event frequency was increased (Figures 6I–J).

### Detection of Two-pore Domain Potassium Channels in Hippocampal Tissue and Hippocampal Cultures

Different potassium channels play a critical role in modulating the excitability of hippocampal networks [57], [58]. We focused on the TREK/TRAAK family of two-pore domain potassium channels with marked temperature sensitivity. The channels TREK-1, TREK-2 and TRAAK all exhibit high open probability at 37°C, which dramatically decreases as the temperature is lowered to room temperature [59], [60]. Moreover, variable levels of mRNA of all three channels have been detected in the mouse hippocampus [61]–[63].

**Figure 7:**
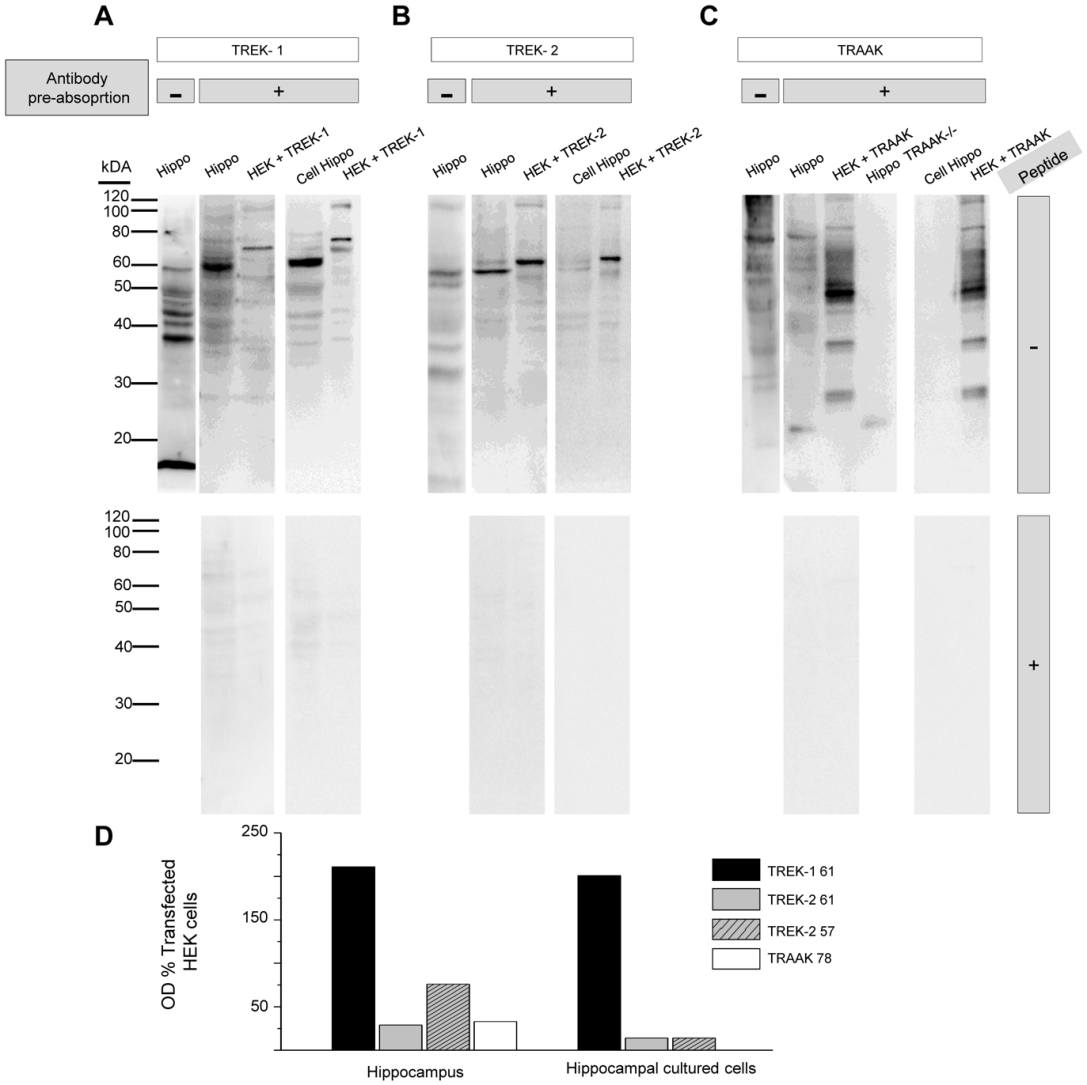
Immunoblot detection of TREK/TRAAK family two-pore domain potassium channels in hippocampal tissue and hippocampal cultures. Western blots of TREK-1, TREK-2 and TRAAK from mouse hippocampus tissue (Hippo), HEK293 cells transfected with TREK-1 (HEK+TREK-1), TREK-2 (HEK+TREK-2), TRAAK (HEK+TRAAK) and hippocampal cultured cells (Cell Hippo). *A*, Upper panel, hippocampus (20 µg of protein) was probed with an anti-TREK-1 antibody with (+) or without (−) pre-absortion with the microsomal fraction of transfected HEK293 cells expressing TREK-2 and TRAAK, as described in Materials and Methods. HEK293 cells transfected with TREK-1 (10 µg of protein) and hippocampal cultured cells (20 µg of protein) were probed with the pre-absorbed anti-TREK-1. Lower panel, the pre-absorbed anti-TREK-1 was incubated with the corresponding antigenic peptide. *B*, Upper panel, hippocampus (20 µg of protein) was probed with (+) or without (−) pre-absortion with microsomal fraction of transfected HEK293 cells expressing TREK-1 and TRAAK, HEK293 cells transfected with TREK-2 (10 µg of protein) and hippocampal cultured cells (20 µg protein) were probed with the pre-absorbed anti-TREK-2. Lower panel, the pre-absorbed anti-TREK-2 was incubated with the corresponding antigenic peptide. *C*. Upper panel, hippocampus (20 µg of protein) was probed with an anti-TRAAK antibody with (+) or without(−) pre-absortion with microsomal fraction of transfected HEK293 cells expressing TREK-1 and TREK-2. HEK293 cells transfected with TRAAK (5 µg of protein) and hippocampal cultured cells (20 µg of protein) were probed with the pre-absorbed anti-TRAAK. Lower panel, the pre-absorbed anti-TRAAK was incubated with the antigenic peptide. *D* The levels of the major bands for each antibody in the hippocampus tissue and hippocampal cultured cells were calculated from the pixel intensity values (minus background) normalized to the pixel intensity values of HEK293- transfected cells and presented as optical density (OD). In this figure same batch of HEK293 transfected cells was used for hippocampus and cultured hippocampal cells, same batch of hippocampus tissue and hippocampal cultured cells were used to probe each antibody. Data correspond to one representative experiment of several experiments using different batches of samples with essentially the same results.

We carried out western blot experiments to determine the presence of different TREK/TRAAK channels in hippocampal primary cultures. As controls, we used adult mouse hippocampal tissue and transfected HEK293 cells. Also, to decrease cross reactivity of the antibodies to other members of the same subfamily of channels, with highly homologous sequences, we used pre-absorption techniques (see Methods). As seen in Figure 7A–C, TREK-1, TREK-2 and TRAAK were detected in lysates from hippocampal tissue, in accordance with previous reports [62], [64], [65]. Only TREK-1 and TREK-2 were detected in our hippocampal cultured cells. In both, hippocampal tissue and cultured cells, the expression of TREK-1 was higher than TREK-2 or TRAAK, in accordance with previous in-situ hybridization reports [63], [65]. Using anti-TREK-1, several bands were observed in mouse hippocampus. Following pre-absortion with microsomal fractions of HEK293 cells transfected with TREK-2 and TRAAK a major band of 61 kDA was apparent both in hippocampus tissue and hippocampal cultured cells (Figure 7A). In HEK293 cells transfected with TREK-1 a major band of 70 kDA was observed. Analysis of the 61 kDA band, normalized to the 70 kDA band of transfected HEK293 cells showed similar intensity in hippocampus and cultured cells (Figure 7D). In competition experiments, after incubating the pre-absorbed antibody with the epitope against which it was raised, no signals to TREK-1 were observed (Figure 7A, lower panel) indicating specific labelling. Anti-TREK-2 labelled several bands in mouse hippocampus tissue (Fig. 7B). When anti-TREK-2 was pre-absorbed with microsomal fraction from HEK293 cells transfected with TREK-1 and HEK293 cells transfected with TRAAK, a major band of 57 kDA and a minor band of 61 kDA were apparent in hippocampus tissue. In HEK293 cells transfected with TREK-2 only the band of 61 kDA was observed. In hippocampal cultured cells, the intensity of the 57 kDA band was greatly reduced, as compared to hippocampus tissue, when it was normalized to the 61 kDA band of transfected HEK293 cells. In competition experiments, after incubating the pre-absorbed antibody with the epitope against which it was raised, no signals were observed (Fig. 7B, lower panel). Finally, when we used anti-TRAAK in hippocampus tissue, it bound to a major band of 78 kDA and minor bands of higher and lower molecular weights. When the hippocampus tissue was incubated with the pre-absorbed anti-TRAAK the major band of 78 kDA and minor bands of 65 kDA, 60 kDA and 20 kDA were apparent. In the hippocampus tissue form TRAAK knock-out mice, only a 20 kDA band was observed. In HEK293 cells transfected with TRAAK a major band of 47 kDA and several minor bands were observed. In hippocampal cultured cells no signals against TRAAK were observed. In competition experiments, after incubating the pre-absorbed antibody with the epitope against which it was raised, no signals were observed either in the hippocampus or transfected HEK293 cells (Fig. 7C, lower panel). The difference between the observed Mr values and the calculated molecular mass, 47 KDa for TREK-1, 59 KDa for TREK-2 and 43 KDa for TRAAK, may be due to different degrees of glycosylation, formation of molecular aggregates or variations in tertiary structure [66]. Figure 7D summarizes the results of relative intensity in immunodetection for the three channels. These results suggest that TREK-1 is the most abundant of the three TREK/TRAAK family channels in hippocampal tissue and in hippocampal cultured cells.

### Two-pore Domain Potassium Channels are Involved in the Cold-evoked Responses

**Figure 8:**
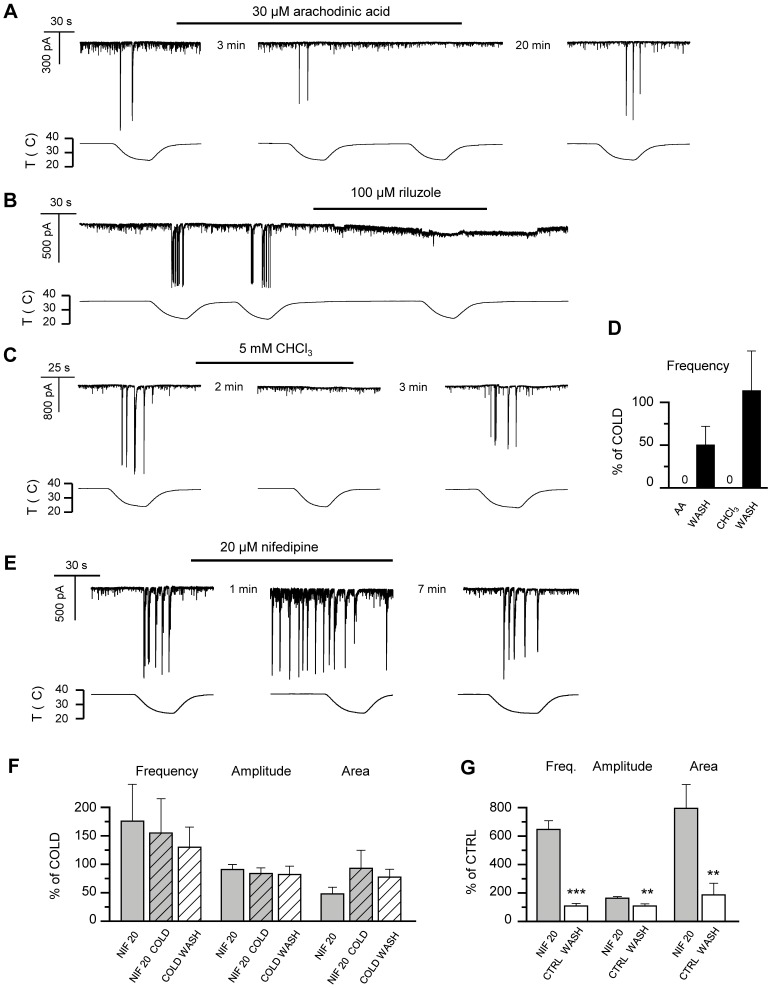
Sensitivity of cooling-evoked responses to arachidonic acid, riluzole, chloroform and nifedipine supports the involvement of TREK channels.

Next, we used pharmacological tools to dissect the contribution of TREK/TRAAK family channels to cold-evoked responses. TREK-1, TREK-2 and TRAAK are all directly activated by polyunsaturated fatty acids, including arachidonic acid, AA [62], [67], [68]. When 30 µM AA was applied to hippocampal cells recorded in whole-cell voltage-clamp mode, baseline synaptic activity remained, but the large, cooling-evoked responses were abolished (Figure 8A). This is consistent with the involvement of thermosensitive TREK/TRAAK channels, which would remain locked into its open state during cooling. In agreement with previous reports [69], we observed a slow onset of AA effects, with a minimum latency of 3 minutes (n = 4; Figure 8A), and even longer times required for washout, consistent with TREK/TRAAK activation. For comparison, a 10°C decrease in the bath solution was effective in only 20 seconds. TREK/TRAAK channels are also activated by the anticonvulsant and neuroprotective agent riluzole [70], [71]. As shown in figure 8B, application of 100 µM riluzole produced a full inhibition of cold-evoked responses (n = 2), consistent with the activation of TREK/TRAAK channels.

Pharmacological tools to distinguish between the three TREK/TRAAK channels are scarce [26], [72]. Nevertheless, TREK-1 and TREK-2, but not TRAAK, are known to be activated by the volatile general anaesthetic chloroform [24], [73]. As seen in Figure 8C, when we applied 5 mM chloroform to the recording chamber, the cooling-evoked activity was reversibly blocked, again consistent with TREK-1 or TREK-2 being prevented from closing. Furthermore, [74], [75] have demonstrated that while 10 µM ruthenium red almost completely inhibits rodent TRAAK channel activity, 20 µM of the compound blocks less than 10% of TREK-1. As described in a previous section (Figure 6I–J), while we observed 10 µM ruthenium red to increase the frequency of both the baseline and the cooling-evoked synaptic activity, it failed to induce the cooling-evoked events at baseline temperature, which would be the expected outcome in the presence of considerable block of background potassium channels. This, together with the positive effect obtained with chloroform, suggests that the observed synaptic responses induced by cooling do not rely on TRAAK activity in any significant degree, and consistent with the lack of expression in our cultures.

Several dihydropyridine Ca^2+^ channel antagonists, including nifedipine and amlodipine, were recently reported to inhibit TREK-1 channels [76]. When we exposed the hippocampal cultures to nifedipine at 20 µM, a concentration estimated to inhibit around 70% of the TREK-1 current, we observed robust activation of transient currents at the baseline temperature, 36–37°C (Figure 8E). Analysis of the whole-cell voltage-clamp recordings revealed that the nifedipine-induced large discharges were very similar to those evoked by cooling, whereas they differed significantly from spontaneous baseline synaptic activity (Figures 8F–G). We also carried out recordings in cell-attached mode and, again, observed nifedipine to evoke large action currents at baseline temperature (not shown). To discriminate between effects of nifedipine on TREK-1 and voltage-gated calcium channels, we also tested the effects of 1 µM nifedipine. This concentration is ineffective in blocking TREK-1 but is several orders of magnitude larger than the IC_50_ for L-type calcium channel block [77]. At 1 µM, nifedipine failed to induce the large responses observed with 20 µM at baseline temperature (data not shown), and also had no effect of the threshold of the cooling-evoked responses (29.8±0.5°C in control *versus* 30.7±0.9°C in nifedipine, p = 0.4; n = 4,) nor on the characteristics of the synaptic activity at baseline temperature, or the cooling-evoked responses. Therefore, these results suggest that the effects of nifedipine are not mediated by inhibition of L-type calcium channels.

We also studied the effect of amlodipine on the hippocampal network. Similarly to nifedipine, 10 µM amlodipine (concentration causing over 90% block of TREK-1 [76] applied at baseline temperature readily evoked similar responses as cooling (not shown). It is worth noting that at this concentration, inhibition of Kv1.4 and T-type Ca^2+^ channel currents is only approximately 10%. In accordance with previous reports [76], we observed the kinetics of inhibition by amlodipine to be slow, taking several minutes to reach steady state and washout.

### Network Activity in Hippocampal Slices is Modulated by Temperature and Two-pore Domain Potassium Channel Modulators

The synaptic connectivity of a cultured hippocampal network is obviously different from that observed in the intact hippocampus. In order to evaluate the possible effects of cooling in the generation of electrical activity in a naturalistic hippocampal network, we recorded spontaneous and evoked activity in postnatal mouse hippocampal slices.

**Figure 9:**
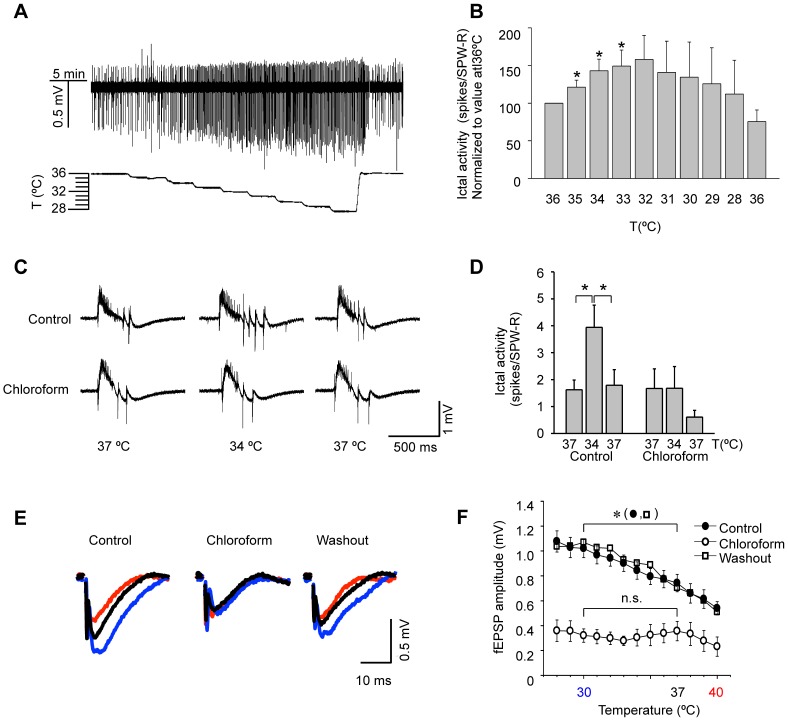
Chloroform blocks the effects of cold temperature on spontaneous ictal activity and evoked field EPSP amplitude in hippocampal slices. *A,* Sample experiment illustrating the increase in CA3 spontaneous ictal activity caused by moderate, progressive cooling, in a disinhibited hippocampal slice. The trace has been filtered at 50 Hz (low-pass). *B*, Averaged values (n = 6 slices) of ictal activity, quantified as number of ictal spikes following each individual SPW-R event, at different temperatures. Values have been normalized to the ictal activity measured at 36°C (*p<0.05, one-way ANOVA). *C,* Sample recordings of SPW-R events followed by ictal activity in a representative experiment before, during and after mild cooling, in control conditions and in 20 mM CHCl_3_. *D,* Summary of ictal activity associated to SPW-R events (n = 11 slices from 7 mice for controls; n = 7 slices from 5 mice for CHCl_3_). Cooling from 37 to 34°C increased ictal activity reversibly and CHCl_3_ abolished such increase. *E*, Representative traces of evoked fEPSPs recorded in CA1 area at 30°C (blue), 37°C (black) and 40°C (red) in control, in 20 mM CHCl_3_ and during washout. Each trace is the average from 5 consecutive fEPSPs. *F*, Average of 5 consecutive traces, illustrating the effect of temperature on fEPSP amplitude. CHCl_3_ (20 mM) applied at 37°C diminished basal fEPSP amplitude and blocked further increases in fEPSP size caused by lowering temperature. (n = 9 slices from 6 mice for control, n = 6 slices from 5 mice for CHCl_3_ and washout). The effects of CHCl_3_ were fully reversible. For clarity, error bars have been removed for the mean values obtained during wash. A two-way ANOVA showed significant differences (p<0.001) between control and CHCl_3_ recordings when including data from all temperature values in the analysis. Posthoc analysis showed significant differences (*p<0.05) between amplitudes at 30 and 37°C in control and wash, with no differences in CHCl_3_.

Spontaneous activity was recorded from the *stratum pyramidale* of the CA3 area in immature (P15–P21), disinhibited slices. Under our experimental conditions (Mg^2+^-free plus 10 µM BMI), all the slices showed some degree of sharp wave-ripple (SPW-R) activity. When present, ictal spikes always followed SPW-R events. Following a baseline period of 10 min at 36°C, we monitored ictal and interictal activity during moderate progressive cooling to 28°C in steps of 1°C, as shown in the experiment illustrated in Fig. 9A. A reduction of just 1 degree Celsius produced a significant increase in ictal activity (quantified as the number of ictal spikes following each SPW-R event). This increase peaked at 32°C, declining progressively at lower temperatures (Fig. 9B). Upon rewarming of the ACSF back to 36°C, ictal activity returned to basal values (Fig. 9A, B). This result reveals that, in disinhibited hippocampal slices, moderate cooling can induce a reversible increase in the ictal activity that resembles, and may be related to the activity induced in hippocampal networks in culture by similar temperature descents.

To evaluate the possible involvement of TREK channels in the increased ictal activity produced by mild cooling, we tested the effect of chloroform (CHCl_3_) (Fig. 9C). The reversible increase in ictal activity produced during cooling from 37 to 34°C was fully blocked by 20 mM CHCl_3_. Under control conditions, ictal activity grew from 1.62±0.36 spikes/SPW-R at 37°C to 3.94±0.83 at 34°C (p<0.05, one-way ANOVA), and this increase was reversible upon rewarwing (Fig. 9D). Application of 20 mM CHCl_3_ to the slices did not alter the extent of ictal activity observed at 37°C. Remarkably, cooling the ACSF to 34°C in the presence of CHCl_3_ failed to evoke the increase in ictal observed previously in the control condition: 1.67±0.73 spikes/SPW-R at 37°C compared to 1.68±0.80 at 34°C (one-way ANOVA, p = 0.99). The effect of CHCl_3_ on ictal activity was poorly reversible (not shown).

We also tested the effect of temperature on evoked synaptic activity in mature (P30–P60) hippocampal slices under normal conditions (i.e. not disinhibited). Extracellular field postsynaptic potentials (fEPSPs) were recorded in *stratum radiatum* of the CA1 area while stimulating Schaffer collateral/commissural afferents. The amplitude of responses were monitored over a wide range or temperatures (Fig. 9E, F). Field EPSP amplitude increased progressively as temperature was lowered, changing from 0.74±0.07 mV at 37°C to 1.02±0.07 mV at 30°C (p<0.01, one-way ANOVA). The increase in amplitude was fully reversible upon rewarming. In the presence of 20 mM CHCl_3_ the fEPSPs at 37°C showed a reduction in amplitude with respect to control values (0.36±0.08 mV). This reduction is consistent with an effect of CHCl_3_ on membrane excitability, because CHCl_3_ would maintain TREK channels in their open state. Moreover, in the presence of CHCl_3_, fEPSP amplitude became insensitive to temperature variations, with a mean amplitude of 0.32±0.05 mV at 30°C (p = 0.72, Two-way ANOVA). The effect of CHCl_3_ on evoked fEPSP was fully reversible upon washout of the drug (Fig. 9E–F).

## Discussion

Temperature changes have important effects on mammalian brain function. We report on a very sensitive excitatory effect of lowering the temperature, within physiological limits of 1–2°C, on synaptic function in networks of cultured hippocampal neurons and hippocampal slices. Network activation occurs in the absence of endogenous expression of the two known cold-activated TRPs (TRPM8 and TRPA1) involved in cold sensing by peripheral sensory neurons, indicating an alternative mechanism for temperature-dependent modulation in central neurons. Our results suggest an important role of thermosensitive TREK/TRAAK channels in the responses observed.

### Moderate Cooling Induces a Novel, Synaptically Mediated, Activity Pattern in Hippocampal Neurons

In our recordings, very subtle cooling of the extracellular medium induces a new form of network activity characterized by large discharges of low frequency and large amplitude. These large synaptic currents include both glutamatergic and GABAergic components. The cooling-evoked activity relies on action potential firing and on synaptic transmission by excitatory glutamate receptors. In line with this, application of CNQX which decreases excitatory, or baclofen which increases inhibitory synaptic transmission, abolished the responses. In the search for the underlying mechanism behind the observed activity, no indication was found to support non-specific actions of temperature on synaptic transmission, such as reduced glutamate re-uptake or the summation of spontaneous mini-PSCs. In addition, the cooling-evoked events exhibited a fixed threshold temperature just below physiological baseline temperature, and are thus not induced by decreasing the temperature from any arbitrary level, suggesting a potentially relevant function *in vivo*.

### Thermosensitive Two-pore-domain Potassium Channels Mediate the Cooling-Evoked Response

We ruled out any decisive role of TRPM8 and TRPA1, cold-activated channels from the transient receptor potential (TRP) family, on cooling evoked network activation. In a previous study, we did not detect these channels in the hippocampal cultures [28]. In a recent publication, Shigetomi et al [78] described a TRPA1-like current in hippocampal astrocytes, but failed to detect the current in hippocampal neurons. Moreover, agonists and antagonists of TRPM8 and TRPA1, neither mimicked nor blocked cold evoked responses. In contrast, the expression and the detailed pharmacological analysis point to temperature-sensitive two-pore-domain potassium channels (K_2p_) as generators of this response. It must be stated that none of the drugs we used targets K_2p_ channels specifically. The lack of specific blockers for most K_2P_ channels is well known in the field [26], [79] and acknowledged in other recent publications characterizing native K_2P_ channels [71]. However, the effects of the different agonists and/or antagonists we used, drugs with different structural profiles, strongly converge on K_2P_ channels, as evidenced by the exclusive identification of K_2P_-related publications when using simple search strategies in Pubmed that combine these terms (e.g. “chloroform” AND “riluzole” AND “ion channel”). In other words, each drug used independently can target different classes of ion channels but, the intersection of 2 or 3 drugs defines a very limited working space centered on K_2P_ channels.

Altogether, despite the pharmacological limitations in distinguishing between the three TREK/TRAAK channels, our experimental data strongly suggest a main role for TREK-1 as the responsible molecular entity behind the cooling-evoked network activation. These functional data were further supported by the strong detection of TREK-1 protein both, in the hippocampal culture and in hippocampal slices. Previous in-situ hybridization results indicate that TREK-1 is highly expressed in hippocampal neurons [61], [63], [80]. Quite remarkably, chloroform, a TREK-1/TREK-2 potassium channel opener, fully suppressed the temperature modulation of ictal activity and evoked CA1 field potentials in hippocampal slices, suggesting a possible role of these channels in the emergence of these activity patterns as well. TREK-1 is highly temperature-sensitive around physiological temperatures, closing rapidly and reversibly with small temperature reductions [59]. It is therefore well tuned to influence the resting membrane potential and neuronal excitability during small temperature changes. However, in blind recordings of hippocampal neurons, the percentage of neurons firing action potentials in response to cooling when synaptic transmission was blocked was very low. This raises the question of how the observed large network discharges are generated. Single neurons are generally recruited to the network activity when they are depolarized to their action potential threshold by accumulation of EPSPs from several presynaptic neurons firing synchronously [81], [82]. It has nevertheless been shown that the firing of a single neuron can activate the entire hippocampal network [81], [83]. This can be achieved if the network is connected through a giant component, i.e. when almost all neuronal subclusters are interconnected through the network [82]. Thus, we find it plausible that despite of TREK-1 being widely expressed in the hippocampal neurons, their closure and subsequent depolarization during cooling may reach firing threshold in only a subset of cells that will propagate the excitation synaptically. Such cells may be rendered more excitable by their recent synaptic/firing history, or by intrinsic determinants (e.g. higher expression of TREK-1, differences in channel modulation).

Our results in slices, showing a progressive enhancement of evoked field EPSP amplitude caused by mild cooling are in accordance with observations by [18] in adult guinea pig hippocampus in which a small decrease in temperature causes an increase in the population spike amplitude and in the number of spikes evoked by current injection. The similar blocking effects caused by chloroform on temperature-modulated fEPSP and on cold-evoked activity in culture suggest a common underlying mechanism, possibly involving two-pore domain potassium channels. It is reasonable to think that the increased synaptic activity caused by cooling in single cells may sum increasing the probability of action potential firing, thus contributing to generation of larger fEPSPs.

### Temperature-evoked and Spontaneous Activity Patterns in Hippocampal Networks

During the first postnatal week, the spontaneous activity in the *in vitro* murine hippocampus is characterized by periodic neuronal discharges, termed giant depolarizing potentials (GDPs) [84], also called early network oscillations [85]. The occurrence of the GDP activity coincides with the temporal window when, due to the reversed chloride gradient, GABA still exerts a mainly excitatory action via GABA_A_ receptors, and glutamatergic signaling is NMDA receptor mediated, while AMPA receptors are relatively quiescent or absent [86]. Consequently, GDP generation has been attributed to the synergistic excitatory actions of GABA_A_ and glutamate (mainly NMDA) receptors [85]–[87]. While a definitive functional significance of GDP-like patterns is yet to be established, it has been postulated that they initially provide a nonspecific signal for growth and maturation via the large rise of [Ca^2+^]_i_, and with the maturation of dendrites and the formation an increasing number of glutamate synapses, GDPs take on a more selective control of synapse formation [87].

There are certain similarities between the neonatal GDPs and the cold-evoked activity patterns observed in the present work. GDPs exhibit a frequency of 0.1–0.3 Hz and a duration of 300–500 ms [86], [87] while we obtained a mean frequency of 0.17±0.08 Hz and a mean duration of 750±160 ms when we analyzed four whole-cell voltage-clamp recordings at a fixed temperature averaging 31.0±0.7°C. Also, both the GDPs and our cooling-evoked events are TTX-sensitive. However, certain discrepancies between the pharmacology of the two activity patterns exist. The hippocampal networks described in this work appear to be in a more mature phase than the time window for GDPs. In our recordings, bicuculline dramatically increased spontaneous network activity indicating that GABA_A_ receptors were mainly inhibitory. Moreover, glutamatergic signaling was largely AMPA receptor dependent, and we observed no spontaneous GDP-like activity. Despite the differing synaptic pharmacology, explainable by the different stage of maturation, it could be hypothesized, that the synaptic connections established during the GDP phase, which decays after the first postnatal week due to the hyperpolarizing (inhibitory) effect of GABA_A_ receptors with the changing chloride gradient, are maintained and can be re-activated through a network-depolarizing stimulus. Such a re-activation of GDP-established connections could provide an example of the transformation of GDPs from growth signals to an activity pattern of the more mature brain.

In the neonatal rat hippocampus *in vivo*, unitary activity occurs in bursts, often associated with sharp waves [88]. These sharp waves were hypothesized to compose the counterpart of the GDPs recorded *in vitro*. Later, it has been shown that sharp waves can be recorded *in vitro* as well [31], [89]. Keeping in mind that in the mature hippocampus, sharp wave activity occurs during slow wave sleep and states of behavioral immobility [32], [90], and that the onset of sleep has been shown to be related to a moderate decrease in body core and brain temperature [7], [91], the novel temperature-related activity described in this work might underlie the emergence of new neural activity patterns observed during physiological temperature fluctuations in the brain. Testing this hypothesis requires additional experiments, in particular recording from the hippocampus *in vivo* during controlled variations in temperature {Leinekugel, 2002 115/id;Mohns, 2008 62 /id}.

### Functional Implications of Cooling-induced TREK Channel Closure in Hippocampal Networks

Previously, TREK-1 activation has been implicated in neuroprotection against damage caused by ischemia and epilepsy [34]. On the other hand, TREK-1 KO mice are resistant to depression [92]. Interestingly, fluoxetine, an antidepressant which reportedly inhibits TREK-1 [93] also reduces brain temperature [94], [95]. In relation with this, Salerian et al [96] proposed therapeutic brain temperature manipulation as a potentially important treatment for mood disorders. Thus, one can anticipate a promising therapeutic potential for temperature-related TREK-1 function in the central nervous system.

### Conclusions and Significance

Our study revealed and unexpected increase in the excitability of mouse cultured hippocampal networks during small temperature reductions from physiological values. Similar changes were observed in the spontaneous neuronal activity and synaptic responses within acute hipocampal slices. The novel activity pattern generated by cooling was independent of thermoTRP channel activity and involved the closure of temperature-sensitive two-pore-domain potassium channels (KCNK), TREK/TRAAK channels. We hypothesize that closure of these background channels may represent a switch mechanism coupling physiological temperature fluctuations with the emergence of state-dependent neural activity patterns.
